# Predictors of Cognitive Decline in Alzheimer’s Disease: A Longitudinal Bayesian Analysis

**DOI:** 10.3390/medicina61050877

**Published:** 2025-05-11

**Authors:** Denisa Claudia Negru, Delia Mirela Tit, Paul Andrei Negru, Gabriela Bungau, Ruxandra Cristina Marin

**Affiliations:** 1 Doctoral School of Biological and Biomedical Sciences, University of Oradea, 410087 Oradea, Romania; miculas.denisaclaudia@student.uoradea.ro (D.C.N.); gbungau@uoradea.ro (G.B.); marin.ruxandracristina@student.uoradea.ro (R.C.M.); 2 Department of Pharmacy, Faculty of Medicine and Pharmacy, University of Oradea, 410028 Oradea, Romania

**Keywords:** Alzheimer’s disease, Bayesian analysis, Reisberg’s GDS, clock-drawing test, Hamilton depression scale, memantine, donepezil

## Abstract

*Background and Objectives*: Alzheimer’s disease (AD) is a progressive neurodegenerative condition that significantly affects cognitive, emotional, and functional abilities in older adults. This study aimed to explore how demographic, clinical, and psychological factors influence the progression of cognitive decline in patients diagnosed with AD. *Materials and Methods:* A total of 101 patients were evaluated retrospectively and followed longitudinally at three different time points, using standardized instruments, including the MMSE, Reisberg’s GDS, clock-drawing test, MADRS, and Hamilton depression scale. Statistical methods included non-parametric tests, mixed-effect modeling, and Bayesian analysis. *Results:* Most patients were older women from rural areas, predominantly in moderate-to-severe stages of AD. Age showed a significant association with cognitive decline (*p* < 0.05), and depression—particularly in moderate and severe forms—was strongly linked to lower MMSE scores (*p* < 0.001). Over 70% of the participants had some degree of depression. The clock-drawing test highlighted visuospatial impairments, consistent with everyday functional loss. Although donepezil and memantine combinations appeared to be more frequently prescribed, no treatment showed a statistically significant advantage, and confidence interval overlaps suggest caution in interpreting differences between therapies. Longitudinal models confirmed a progressive and accelerated decline, with inter-individual variability becoming more pronounced in later stages. Although comorbidities, such as hypertension and diabetes, were frequent, they did not show a statistically significant effect on MMSE scores in this cohort. *Conclusions:* Age and depression appear to play central roles in the pace of cognitive deterioration in AD. Given the limited efficacy of most current therapies, these findings highlight the need for earlier intervention and a more personalized, integrated approach—combining cognitive care, psychiatric support, and comorbidity management—to better meet the needs of patients with AD.

## 1. Introduction

Alzheimer’s disease (AD) is a major challenge for healthcare systems, having a progressive evolution combining cognitive impairment and severe psychosocial damage. AD is a chronic progressive disease that affects people over 65 years old worldwide. It starts with pathophysiological alterations in the brains of those who are afflicted, years before any clinical symptoms appear [1].

AD’s clinical symptoms underlying the pathophysiological process are best understood as a continuum: patients have mild, moderate, and severe dementia related to AD after progressing from normal cognition to mild cognitive impairment (MCI) caused by AD [2].

These pathophysiological alterations include the buildup of toxic amyloid-β (Aβ) species, the formation of hyper-phosphorylated Tau protein neurofibrillary tangles, and neurodegeneration, which could be brought on by the brain’s microglia activating uncontrollably and secreting inflammatory and neurotoxic substances [3]. Those who have such alterations may not show any symptoms at all or may show clinical signs that range from little memory loss to severe incapacitating cognitive and memory impairments. Other neuropsychiatric symptoms may appear as AD worsens, such as mood swings, aggression/agitation, confusion, disorientation, and, in later stages, delusion/hallucination [4,5].

Several clinical measuring scales that primarily assess a patient’s level of cognitive impairment have been used for decades for classifying AD patients. Rating scales are crucial instruments for the diagnosis, staging, evaluation, and thorough observation of AD symptoms, as well as for assessing the results of treatments. Because cognition is the primary symptom of AD, it was the focus of most AD examinations for many years. Currently, conventional outcome measures in AD clinical trials include rating scales for evaluating a patient’s overall impression, behavior, and functioning in addition to cognitive status. Additionally, measures for evaluating patients with advanced AD have been created [6].

The total evaluation of disease progression in ordinary medical practice is still time consuming and complex, even with the substantial development of grading scales for AD research. One of the reasons for this is that evaluating all the symptom domains related to AD requires the use of a set of scales, which is typically a laborious process for both the doctor and the patient/caregiver. Furthermore, the majority of the rating scales do not apply to all phases of AD severity; in other words, many assessment instruments are not reliably sensitive enough to gauge the course of the disease or the impacts of treatments on the entire patient group [7].

Important illness prognostic indicators, like the existence of concurrent disease conditions, are not taken into account in the current classification system. Comorbid disorders that arise either concurrently with or before AD may have impacts on the disease’s overall clinical state and progression. AD has been linked to well-known chronic illnesses, such as diabetes [8,9], heart disease [8], depression [10], and inflammatory bowel disease, according to multiple lines of evidence [11]. Coexisting medical issues may eventually negatively affect how AD sufferers manage their illness.

Because of pathological mechanisms that may be shared by certain comorbidities and AD, such as the accumulation of amyloid-beta (Aβ) and the presence of the APOE ε4 allele, there may be modifications to the clinical progression of AD. Inconclusive results and a range of positive, negative, and neutral relationships between comorbidities and AD have frequently resulted from inconsistent study designs and methods [12,13].

Although some studies have demonstrated that diabetes mellitus increases the risk of AD, to the extent that AD is referred to as “type 3 diabetes”, other studies have found no discernible changes in risk. This could be because of different design factors, such as the population, sampling techniques, and the various forms of diabetes that were examined [14,15].

In previous studies, comorbid conditions, like stroke, falls, and depression, were found to have negligible correlations with AD. Some of these comorbidities have mixed relationships in the literature, indicating that the inconsistencies in these studies are caused by different populations, different techniques, and the complexity of some comorbidities (e.g., treatment effects and subtypes) [16,17]. Depression may be a prodromal symptom of AD and a risk factor according to some research [18], while other investigations have claimed that depression is a prodromal sign [19].

Although multiple clinical and demographic characteristics have been previously investigated in the context of AD, their complex interactions and combined effects on disease progression remain insufficiently explored. Using advanced statistical modeling, this study analyzed the evolutions of cognitive performance (via the MMSE) and functional impairment (via the Reisberg scale and clock test) in relation to demographic variables, the severity of the depression (assessed using the MADRS and Hamilton scales), the presence of comorbidities, and the impact of anti-AD pharmacological therapy (memantine, donepezil, and rivastigmine).

## 2. Materials and Methods

### 2.1. Study Design

This observational, retrospective, longitudinal study enrolled a cohort of 101 individuals with a principal or secondary diagnosis of AD, selected from the database of the Psychiatry Department at Bihor County Emergency Clinical Hospital in Oradea, Romania, in the period October 2022–December 2023. In order to be enrolled in the study, the patients should have met the following inclusion criteria: a confirmed diagnosis of AD, according to NIA-AA criteria, regardless of the evolutionary stage, with complete medical data, which allow the analysis of sociodemographic and clinical factors relevant to the study, as well as the tracking of the evolution at three different moments in time (only patients who had data for at least three evaluations, with the same therapeutic regimen, were included). A diagnosis of other forms of dementia (e.g., vascular or frontotemporal dementia), major psychiatric disorders (e.g., schizophrenia or bipolar disorder), a recent history of substance abuse (within the past 12 months), incomplete medical records, and severe concomitant medical conditions that could influence the results were considered as exclusion criteria. Clinical data were collected from medical records; data obtained using standardized assessment tools, such as the MMSE (mini-mental state examination), MADRS (Montgomery–Åsberg depression rating scale), clock test, and Hamilton rating depression scale, were centralized to measure the severity of the cognitive and depressive symptoms of the patients. Data on pharmacological therapy were also collected, for analyzing its impact on the evolution of the disease. Subsequently, the data were analyzed using descriptive and inferential statistical methods to determine significant correlations between the studied variables. To track the development of the parameters of interest, 303 observations were made, with each patient being assessed three times.

This research was approved by the Research Ethics Committee of the Faculty of Medicine and Pharmacy of Oradea at the University of Oradea (no. CEFMF/02, 30 September 2021) and by the Ethics Committee of the Oradea County Emergency Clinical Hospital (No. 38918, 1 November 2021) and was conducted in compliance with the principles of the Declaration of Helsinki regarding studies on human subjects.

### 2.2. Instruments and Variables

In Table 1, there are described the standardized tests designed for monitoring cognitive performance and depression symptoms, used as instruments for evaluating the studied cohort.

Patients’ comorbidities have also been used as cohort evaluation variables: hypertension, diabetes, depression, obesity, dyslipidemia, and anemia; their demographic characteristics, as independent variables, are considered as predictors, including age, gender, educational level, place of residence. The anti-AD type of therapy (memantine, donepezil, or rivastigmine) was also evaluated for assessing its impact and effectiveness on the progression of the disease.

### 2.3. Statistical Analyses

Statistical analyses were performed using R programs, version 4.4.0 Copyright (C) 2024, the R Foundation for Statistical Computing, R Core Team (2024), Vienna, Austria. URL https://www.R-project.org, with the following additional packages: gtsummary, lme4, lmerTest, and sjPlot [25].

To analyze the progression of the cognitive decline, as measured based on MMSE scores, we employed a mixed-effect linear regression model. This approach was chosen because of the longitudinal nature of the data (repeated measures across three time points) and the need to account for both within-subject and between-subject variabilities. The model included time, sex, age, depression severity (MADRS), educational level, anxiety (Hamilton), type of pharmacological therapy, and the use of psychotropic co-medications (e.g., antidepressants, anxiolytics, antipsychotics, or sedatives) as fixed effects. By including these covariates, the model allowed us to assess the independent effect of each predictor while adjusting for potential confounders. In particular, it enabled us to examine whether observed gender differences in MMSE scores were confounded by educational attainment or depressive symptom severity. Random intercepts for each patient were included to capture individual baseline cognitive levels and trajectory differences over time. This multilevel structure offers a robust way to reduce the risk of allocation bias, particularly in observational data, where the treatment was not randomized. In addition, we explored interaction terms, such as age × depression severity and gender × educational level. These interactions, however, did not reach statistical significance and were excluded from the final model to maintain clarity of interpretation and model parsimony, especially considering the sample size. All the model assumptions (e.g., residual normality) were verified and met, and diagnostic checks confirmed a satisfactory model fit.

For the Reisberg and clock scores, which are ordinal variables (with ordered values but not equal distances between categories), we employed Bayesian ordinal regression models with mixed effects. The choice of this method was justified by the fact that conventional frequentist models often face limitations when the variables are ordinal and when the predictors are correlated with each other. Bayesian modeling also allows the incorporation of prior information while maintaining flexibility in estimation when standard assumptions are not met. The models included random intercepts for each patient to account for inter-individual variability, and priors were selected to be weakly informative in order to improve convergence without exerting undue influence on the posterior distributions. Specifically, fixed intercepts followed a normal (0, 3) distribution, predictor coefficients were assigned Student-t (7, 0, 2) priors, and random intercepts were modeled using Student-t (3, 0, 2.5) priors. The number of intercepts was determined by the number of ordinal levels minus one: six thresholds for the Reisberg scale (which has seven stages) and three for the clock-drawing test (with four ordered categories). This is standard practice in cumulative ordinal regression and ensures appropriate modeling of the ordered transitions between outcome categories. To support the robustness of the prior structure, we performed a prior sensitivity analysis by initially fitting the models using the default priors from the brms package (flat priors for coefficients and broader Student-t distributions for intercepts). These default settings yielded wider posterior distributions and less stable convergence indicators (e.g., slightly elevated $\hat{R}$ and lower n_eff values). The final chosen priors led to more reliable parameter estimation and convergence. In addition, no divergent transitions were observed during MCMC sampling, and all the models met convergence criteria ($\hat{R}$ = 1, high n_eff). The model’s performance was further assessed using leave-one-out cross-validation (LOOCV) with metrics such as ELPD, p_loo, and LOOIC to evaluate both the fit quality and model complexity.

## 3. Results

### 3.1. Sociodemographic and Clinical Characteristics

The study included 101 patients diagnosed with AD, with an average age of 75 years (SD ± 9), ranging from 61 to 90 years. The majority were female (77%), while 23% were male. More than half of the patients resided in rural areas (56%), with the remaining 44% in urban settings. Regarding education, 63% of the patients had attended secondary school, while 19% had no formal education, and 18% had only a primary education. These demographic characteristics are detailed in Table 2.

Regarding pharmacological treatment, the most frequently used medication was memantine (31.68%), followed by donepezil (23.76%) and rivastigmine (11.88%). A subset of patients received combination therapies, including memantine with donepezil (23.76%) or memantine with rivastigmine (11.88%). Four patients (3.96%) were not receiving any anti-AD medication at the time of the study (Table 2).

Cognitive impairment was assessed using the mini-mental state examination (MMSE) and the Reisberg global deterioration scale (GDS). The mean MMSE score was 18.6 (SD ± 5.6), indicating moderate cognitive impairment in most patients. According to the MMSE classification, 38% had mild impairment (MMSE > 20), 44% had moderate impairment (MMSE 10–20), and 18% had severe impairment (MMSE < 10). The Reisberg scale provided additional insight into disease severity: 10% of the patients were classified as Stage 3 (mild cognitive impairment), 32% as Stage 4 (moderate cognitive decline), 41% as Stage 5 (moderate–severe cognitive decline), and 17% as Stage 6 or 7 (severe impairment). These findings suggest that most patients were already experiencing significant cognitive deterioration at the time of the assessment (Table 3).

The assessment of spatial orientation abilities, performed using the “clock-drawing” test, showed that most patients (42%) fall into stage II, indicating mild-to-moderate deficits, while 18% present severe alterations (stage IV).

In terms of depressive symptomatology, the assessment on the MADRS scale showed that approximately one-third of the patients (29%) did not present any significant depressive symptoms, but the rest of the patients are mainly distributed in stages I and II of depression severity (38% and 16%, respectively), and 18% experienced severe forms (stage III). The Hamilton scale confirmed the presence of affective disorders, with 31% of the patients having moderate depression (stage II) and 24% having severe depression (stage III). These results highlight not only the variable severity of the cognitive impairment but also the high frequency of depression associated with AD, an essential aspect for understanding the complexity of the management of these patients.

### 3.2. Cognitive Decline and the Evolution of MMSE Scores in Alzheimer’s Disease

During the follow-up, the patients showed significant and consistent cognitive declines, reflected by decreases in MMSE scores. From an initial mean score of 18.56 points (95% CI: 17.47–19.66), the values decreased to 17.67 points at the first reassessment (*p* < 0.001) and subsequently to 16.60 points at the end of the follow-up period (*p* < 0.001) (Table 4).

**Figure 1:**
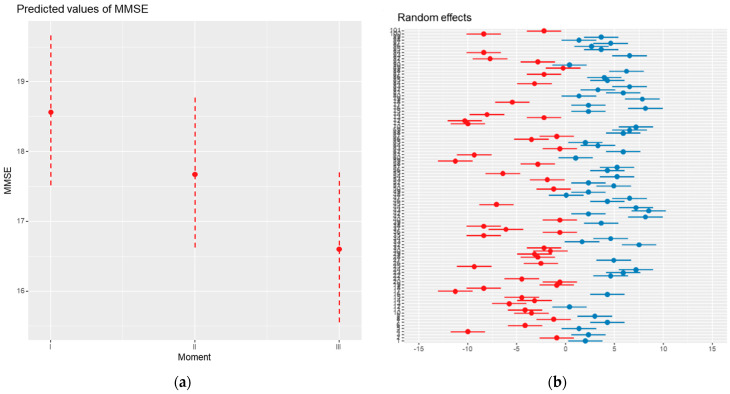
MMSE evolution during the studied period: (**a**) MMSE moment effect graph; (**b**) MMSE moment R effect graph. (**b**) each point represents the estimated patient-specific effect (e.g., deviation from the population mean), and horizontal lines indicate the 95% confidence interval. Red points correspond to negative estimated effects and blue points to positive ones. Because of the high number of patients, Y-axis labels (patient identifiers) may overlap. All estimates remain distinct and interpretable.

These findings were validated by hierarchical linear analysis, which also showed a significant inter-individual variability (ICC = 0.92) and the statistical significance of the cognitive deterioration. According to the model, individual differences accounted for a significant amount of the overall variation in MMSE scores (conditional R^2^ = 0.922), as Figure 1 illustrates.

### 3.3. Analysis of Predictive Factors for Cognitive Decline

#### 3.3.1. Influences of Sociodemographic Factors and Clinical Conditions

The hierarchical linear model applied to patients with AD showed that among the sociodemographic factors, age and gender are the main factors influencing MMSE scores (Table 5). Specifically, as patients age, MMSE scores decrease, on average, by approximately 0.13 points for each additional year (*p* = 0.026), which reflects the negative impact of age on cognitive function. Also, male patients obtained, on average, MMSE scores higher by 2.56 points compared to those of female patients (*p* = 0.046), suggesting that cognitive decline may be less pronounced in men. In contrast, other variables analyzed, such as the educational level, residential environment, or type of diagnosis, did not have a significant influence on the cognitive performance.

**Figure 2:**
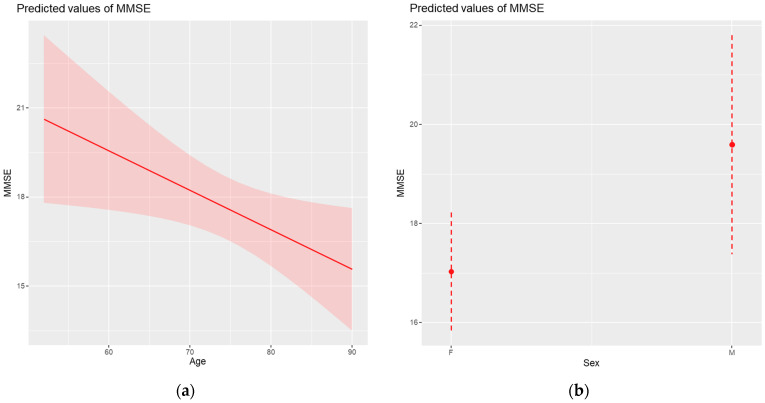
The significant relationships between MMSE scores and (**a**) age and (**b**) gender.

Figure 2a illustrates the relationship between age and MMSE scores, with a clear trend of decreasing scores with increasing age. Figure 2b shows the differences in MMSE scores between men and women. The results confirm that men score higher, on average, than women, the difference being statistically significant. Although the coefficients for age (β = −0.13) and sex (β = +2.6 for males) reached statistical significance, the magnitude of these effects is relatively modest from a clinical perspective. An annual decrease of 0.13 MMSE points, or a 2.6-point average difference between the sexes, does not suggest major functional changes and should be interpreted in the context of the overall cognitive variability and the natural progression of AD.

The analysis performed to evaluate the impacts of different clinical conditions on MMSE scores is presented in Table 6. Although some common conditions, such as hypertension and diabetes, were present in the cohort, their estimated effects on MMSE scores were generally small and accompanied by wide confidence intervals. This indicates a high degree of uncertainty and suggests that at this sample size, these conditions may have a limited or undetectable influence on cognitive performance.

Depression severity proved to be an important factor associated with cognitive performance (Table 7). The relationship between depression and MMSE scores was assessed using both the MADRS and Hamilton scales.

**Figure 3:**
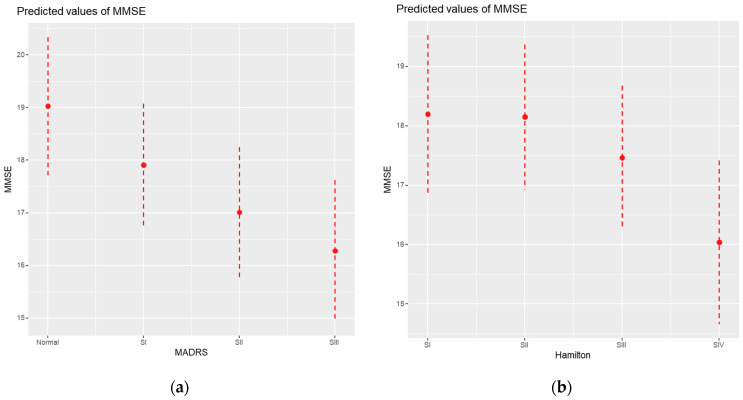
(**a**) MMSE-MADRS effect graph; (**b**) MMSE–HAMILTON effect graph.

According to the MADRS results, a significant and progressive association was observed between depression severity and lower MMSE scores. Patients with mild depression (Stage I) had MMSE scores lower by 1.12 points (*p* = 0.027); moderate depression (Stage II), by 2.01 points (*p* = 0.001); and severe depression (Stage III), by 2.75 points (*p* < 0.001) compared to non-depressed patients. Marginal means confirmed this pattern (Table 8; Figure 3a), showing lower cognitive performance as depression worsened.

Using the Hamilton scale, only severe depression (Stage IV) was significantly associated with a reduction of 2.16 points in MMSE scores (*p* = 0.002), while mild and moderate stages were not statistically significant predictors (Table 8; Figure 3b). Despite slight differences between the two scales, both confirmed the negative impacts of moderate and severe depressive symptoms on cognition.

Both models explained most of the MMSE variance through inter-individual variability (ICC ≈ 0.90), while the direct contribution of depression was modest but consistent (marginal R^2^: 1.7–2.7%).

Given the significant association between depression severity and cognitive performance (MMSE), as indicated by the MADRS scale (*p* < 0.001) and only partially by the Hamilton scale (significant for Stage IV only, *p* = 0.002), we further assessed the correlation between the two instruments at the baseline (Moment I). The Spearman correlation coefficient (ρ = 0.106; *p* = 0.289) revealed a weak and statistically non-significant relationship, suggesting that although both scales capture depressive symptoms, they may reflect distinct dimensions of depression. This observation is consistent with the patterns observed in the regression models.

Although the *p*-values indicated a statistically significant association between depression severity and lower MMSE scores, the observed effect sizes were relatively modest from a clinical standpoint. For instance, MMSE reductions ranged from 1 to 2.7 points, depending on the depression stage (MADRS), or up to 2.16 points for Stage IV on the Hamilton scale. These differences may not necessarily reflect major impairments in daily functioning. They should be interpreted in light of individual variability and the expected progression of AD. Nevertheless, the consistency of these associations across multiple models and time points supports the clinical relevance of depression as an aggravating factor in cognitive decline.

#### 3.3.2. Impacts of Therapies on MMSE Scores

The analysis of the effectiveness of anti-AD treatments (Table 9) indicated that most of the drugs used were not associated with significant improvements in MMSE scores. The administration of memantine, regardless of the dose, did not produce any notable changes in cognitive performance (*p* = 0.814). Similarly, neither donepezil nor rivastigmine, regardless of the dose administered, has any significant impact on the evolution of MMSE scores. Psychotropic medications, including antidepressants, anxiolytics, and antipsychotics, were also included in the model as covariates. None of these treatments was significantly associated with changes in MMSE scores, suggesting that their roles in modulating cognitive performance were limited in this population.

#### 3.3.3. MMSE Evolution over Time According to Therapy

The evolution of MMSE scores over the three assessment times (Table 10) showed that patients without therapy started with higher scores (23.75 points) and experienced moderate declines at subsequent assessments (21.75 points at the final time point). In contrast, patients in the treatment groups had lower scores from the beginning, with a similar trend in deterioration over the following time points. Although the donepezil (CII) and memantine + rivastigmine (CV) groups showed more pronounced decreases, the wide overlap of the confidence intervals indicates a high level of uncertainty in the estimated effects. In this context, the lack of a statistical significance does not rule out potentially meaningful clinical effects, but it does underscore that the available data were insufficient to support definitive conclusions about the relative efficacies of these treatments.

#### 3.3.4. The Final MMSE Model (Significant Predictors)

In the final mixed-effect model, we accounted for predictors including age, gender, depression severity, and therapy. To better isolate treatment effects and minimize the influence of baseline cognitive differences, the initial MMSE scores were examined across therapy groups and considered in the model interpretation. Depression, particularly in moderate and severe forms, continued to be the primary predictor linked to lower MMSE scores, according to the final mixed model (Table 11). Only the memantine + rivastigmine (CV) combination was linked to a significant extra drop in the MMSE score among the treatments (β = −6.2, *p* = 0.037). On the other hand, neither gender nor age had a significant impact on how the scores changed in this model. Although male patients initially appeared to perform better on the MMSE, this difference diminished in the final model after adjusting for age and depression severity. These findings suggest that the observed gender disparity may be driven more by clinical factors, particularly a higher prevalence or intensity of depressive symptoms among women, than by gender itself.

Using a backward selection procedure, we developed a parsimonious model that retained only predictors with statistically significant associations (*p* < 0.05). The analysis revealed that age was independently associated with lower MMSE scores, even after adjusting for depression severity. Specifically, each additional year of age was linked to a mean decrease of 0.12 points in the MMSE score (β = −0.12, 95% CI: from −0.24 to 0.00, *p* = 0.041).

Depression severity, assessed using the MADRS scale, also showed a clear negative association with cognitive performance. Compared to patients without depression, those with mild depressive symptoms scored, on average, 1.1 points lower on the MMSE (β = −1.1, 95% CI: from −2.1 to −0.11, *p* = 0.030), those with moderate symptoms scored 2.0 points lower (β = −2.0, 95% CI: from −3.1 to −0.83, *p* < 0.001), and those with severe symptoms scored 2.7 points lower (β = −2.7, 95% CI: from −4.0 to −1.4, *p* < 0.001).

Pharmacological therapy, on the other hand, did not emerge as an independent predictor of MMSE scores in this model. This finding supports the earlier observation that the effects of drug treatments may be modest compared to the influences of age and depression severity. The results are summarized in Table 12, highlighting the cumulative impact of aging and depressive symptoms on cognitive decline.

**Figure 4:**
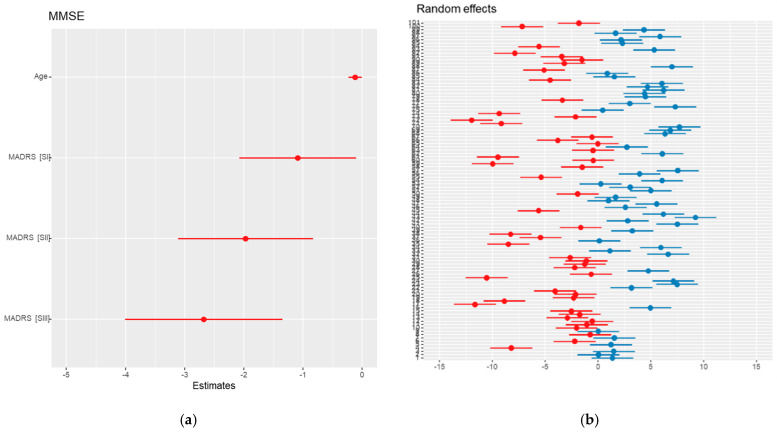
Effects of predictors associated with MMSE scores: (**a**) MMSE multiple effect; (**b**) MMSE multiple R effect. (**b**) each point represents the estimated patient-specific effect (e.g., deviation from the population mean), and horizontal lines indicate the 95% confidence interval. Red points correspond to negative estimated effects and blue points to positive ones. Because of the high number of patients, Y-axis labels (patient identifiers) may overlap. All estimates remain distinct and interpretable.

Although the proportion explained by fixed effects (treatment, depression, age, and sex) was small (marginal R^2^ = 6.3%), the model explained 89% of the total variability in MMSE scores (conditional R^2^ = 0.898), indicating that patient differences continued to be the primary factor in the variability of the cognitive function (Figure 4). Moreover, although the average clinical effects for variables such as age or depression severity were statistically significant but modest, the substantial variance captured by random effects suggests that for certain individuals, the cognitive changes may be clinically meaningful.

### 3.4. Bayesian Analysis of Factors Associated with the Evolution of the Reisberg Score

**Figure 5:**
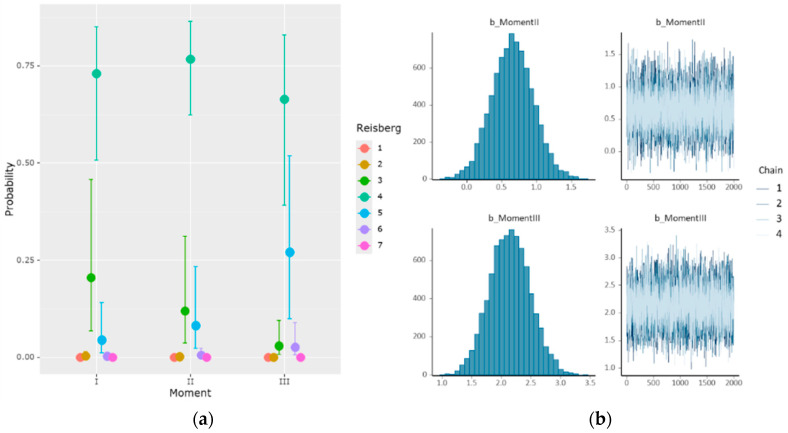
Bayesian mixed-effect ordinal regression model: (**a**) the estimated effect of the time on the Reisberg scale; (**b**) the distribution and uncertainty of the effects.

The Bayesian ordinal regression model identified several relevant factors in the progression of the AD severity, as assessed based on the Reisberg scale. The results showed a clear upward trend in Reisberg scores over time, particularly in later stages of the disease, with significant transitions between thresholds 4 → 5, 5 → 6, and 6 → 7 (Table 13, Figure 5).

**Figure 6:**
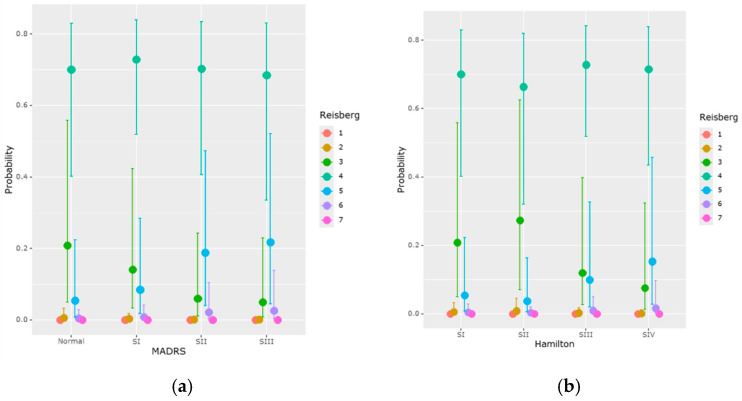
Effect of depression on the Reisberg score: (**a**) MARDS; (**b**) Hamilton.

Higher Reisberg scores were significantly associated with moderate and severe depression (as measured based on the MADRS scale) among the clinical characteristics, suggesting a more marked decrease in cognitive function in these individuals. In particular, as seen in Table 14 and Figure 6, mild depression raised the Reisberg score by 1.45 points (95% CI: 0.20–2.73), whereas severe depression raised it by 1.65 points (95% CI: 0.23–3.12). Mild depression (MADRS SI), on the other hand, exhibited no significant impact (the credibility interval included the value 0). However, neither gender nor age showed any significant correlation with the Reisberg score’s progression; the confidence intervals for these variables contained the value 0.

**Figure 7:**
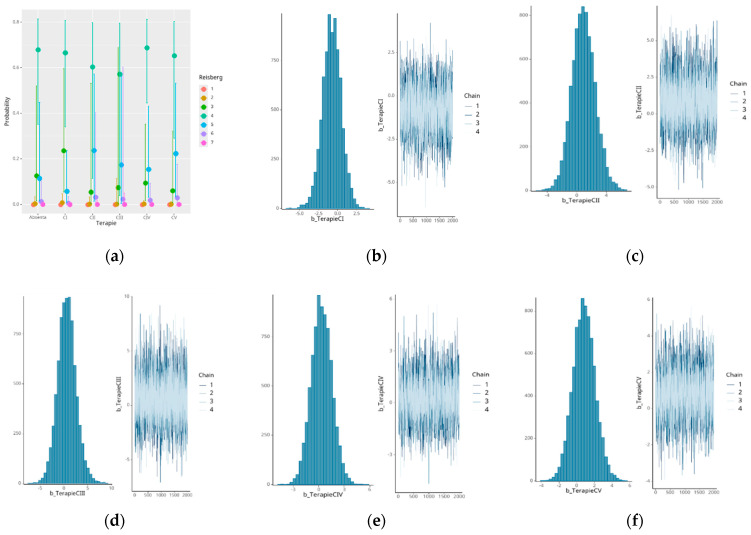
Effects of different therapies on the Reisberg score: (**a**) No therapy, (**b**) therapy CI, (**c**) therapy CII, (**d**) therapy CIII, (**e**) therapy CIV, and (**f**) therapy CV.

None of the studied therapeutic regimens (memantine, donepezil, rivastigmine, or their combinations) significantly affected the Reisberg score’s evolution in terms of the pharmacological effect (Table 15, Figure 7). There was a considerable degree of uncertainty in the calculated effects because all the confidence intervals contained the value 0. These findings highlight that the wide credibility intervals for treatment effects, particularly for combination therapies, such as memantine + rivastigmine, reflect substantial uncertainty rather than systematic differences. Thus, conclusions regarding the comparative effectiveness of treatments should be considered as exploratory and interpreted with caution.

To evaluate the predictive accuracy and stability of the Bayesian ordinal regression model applied to the Reisberg score, we used leave-one-out cross-validation (LOOCV). The model’s performance was quantified using standard metrics: expected log pointwise predictive density (ELPD), effective number of parameters (p_loo), and the leave-one-out information criterion (LOOIC). These results (Table 16) indicated a good fit with moderate model complexity. Additionally, we compared the full model to simplified alternatives to assess the relative importance of the key predictors. The comparison confirmed that depression severity, especially moderate and severe levels (MADRS SII and SIII), was the strongest and most consistent predictor of cognitive decline, while age, gender, and therapy showed limited or uncertain contributions.

### 3.5. Additional Analysis of Clock Scores with the Bayesian Model

The Bayesian ordinal regression model applied to the clock scores revealed a significant and progressive decline in the visuospatial function over time. Specifically, both follow-up moments were associated with lower scores compared to those at the baseline: Moment II (β = −1.15, 95% CI: from −1.86 to −0.45) and Moment III (β = −1.38, 95% CI: from −2.13 to −0.67) (Table 17). These findings confirm a consistent deterioration across time points, highlighting the utility of the clock-drawing test in monitoring functional decline in AD.

**Figure 8:**
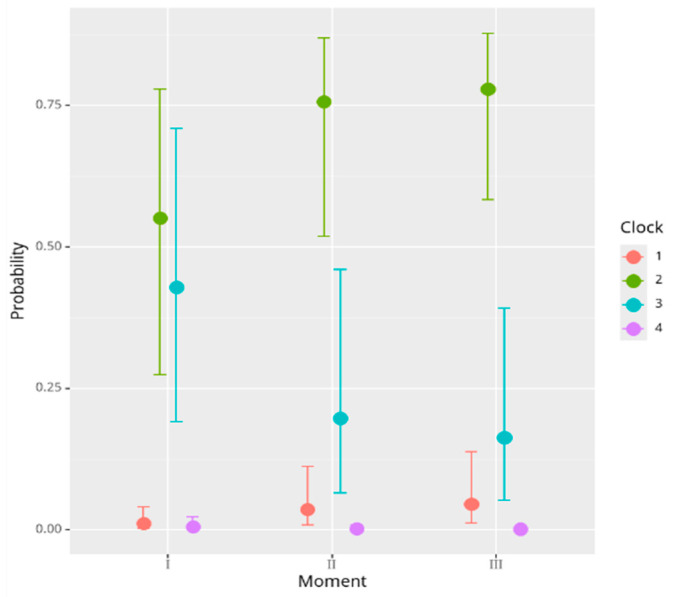
Evolution of the clock score over time.

The ordinal thresholds (intercepts) increased progressively (from −4.46 to 5.20), indicating appropriate separation between the stages of impairment (Figure 8).

Only the male gender, however, had a significant positive correlation with clock scores in the enlarged model (Table 18), while other factors, such as anxiety (Hamilton) and depression (MADRS), did not consistently affect the outcomes.

Additionally, the predictive performance of the Bayesian model applied to the clock score was assessed using leave-one-out cross-validation (LOOCV). The results (Table 17) indicated a reasonable model fit (elpd_loo = −228.4, p_loo = 86.9, LOOIC = 456.7) with moderate model complexity, supporting the robustness of the findings.

When comparing the full model to a simpler model based solely on sex as a predictor, the difference in ELPD was small (−4.4, SE = 2.6) (Table 19). This relatively modest difference suggests that adding clinical factors did not substantially improve the predictive performance compared to that of the simpler model. Thus, although the models confirmed the downward trend in clock scores over time, the clinical relevance of the additional predictors appeared to be limited within this sample.

## 4. Discussion

The number of AD diagnoses continues to rise, particularly among the elderly, because of a combination of genetic, lifestyle, and environmental factors. By 2025, the World Health Organization estimates a 14% increase in AD cases, mostly driven by aging populations [26].

In our study, most patients were elderly women from rural areas, with a secondary education, aligning with prior research that suggests a higher prevalence of AD in women, likely because of their longer life expectancy and hormonal factors [27].

The most used treatment in the studied group involved memantine alone or in combination with other drugs (donepezil and rivastigmine). Memantine is an N-methyl-D-aspartate (NMDA) receptor antagonist used to manage moderate-to-severe AD. It works by regulating glutamate activity, which can help to protect neurons from excitotoxicity—a key mechanism of neurodegeneration in AD [28]. The high proportion of participants on memantine underlines the importance of this drug as a cornerstone in the treatment of AD patients, likely targeting moderate-to-severe cognitive decline. The other two most used drugs, donepezil and rivastigmine, are both cholinesterase inhibitors administrated in patients with mild-to-moderate AD. The data in this study reflect current clinical practice trends aimed at addressing multiple pathological pathways in AD [29].

When analyzing the cognitive and psychological characteristics of patients, the outcomes showed a cohort with varying degrees of cognitive impairment and associated psychiatric symptoms, evaluated using tools like the MMSE, Reisberg global deterioration scale (GDS), clock-drawing test, MADRS, and Hamilton depression scale. The predominance of moderate-to-severe AD (per the MMSE and GDS) aligns with the high use of memantine and combined therapies (e.g., memantine + cholinesterase inhibitors), which are guideline-recommended for advanced stages [30]. The clock-drawing test results highlight functional impairments that affect daily living (e.g., dressing and navigation), reinforcing the need for caregiver support. Visuospatial impairments detected in the clock-drawing test are consistent with findings in other studies, where this test is used to evaluate parietal lobe dysfunction in AD [31].

Depression rates in this cohort also align with findings from other studies, indicating that up to 50% of AD patients experience depressive symptoms. The MADRS and Hamilton scales are validated tools for assessing depression severity in dementia populations [32]. The Alzheimer’s Association emphasizes using comprehensive cognitive assessment protocols during wellness visits to detect dementia early [33]. But recent studies include digital tools for preclinical AD detection, which focus on subtle cognitive changes associated with biomarkers, like amyloid-beta (Aβ). These tools may complement traditional assessments, like the MMSE, in future research [34].

In our study, there is a noticeable decline in MMSE scores over time, which underscores the importance of ongoing monitoring and potentially adjusting treatment strategies to address worsening symptoms. The MMSE scores at all the time points indicate moderate cognitive impairment, demonstrating a progressive worsening of cognitive function consistent with the expected progression of AD or similar neurodegenerative conditions. Research shows that individuals with AD often see a 2–4-point-per-year reduction in their MMSE scores. Although the precise rate may vary depending on individual characteristics and the illness stage, the observed reduction in this research is consistent with these assumptions [35,36].

The analysis of the predictors showed a significant association between age and cognitive decline (*p* < 0.05), with each additional year being associated with a mean reduction in cognitive performance. This finding is consistent with the those in the literature, which indicates that aging causes progressive cognitive decline, especially in the context of AD. The major risk factor for developing AD is living longer; AD prevalence doubles every 5 years after age 65 and approaches 50% by age 85 [37]. Thus, early identification and early intervention are essential to slow this decline.

The educational level and living environment were not major determinants of disease progression in our cohort, likely because of the homogeneity of our sample. Prior meta-analyses have indicated mixed findings for education’s role in dementia risk, with significant variations based on geographic and socioeconomic contexts. Although some studies highlight the protective effect of the educational level [38], our findings suggest that individual cognitive resilience and other lifestyle factors may be more influential.

Comorbidities, such as hypertension, diabetes, cerebral atrophy, and depression, were the most common in our cohort, consistent with previous studies identifying cerebrovascular disease, metabolic disorders, and obesity as key risk factors for AD [39,40]. Metabolic and cardiovascular comorbidities, although prevalent, did not significantly accelerate cognitive deterioration in our sample. Previous research has indicated that multimorbidity in AD patients increases care demands and hospital readmissions, emphasizing the importance of comprehensive management [41]. Studies have shown that vascular and metabolic conditions contribute to AD risk, particularly in patients with hypertension, diabetes, and obesity, highlighting the need for early intervention in these populations [42,43]. Analyzing the impacts of medical conditions on AD patients’ MMSE scores, none of the conditions showed any strong, statistically significant association. Many recent studies have found stronger associations, especially with brain atrophy, diabetes, hypertension, and dyslipidemia. The variability in these findings could be because of differences in the study design, sample sizes, and population characteristics [44,45,46]. The absence of significant associations between comorbidities and MMSE scores may reflect both the relatively small sample size and the retrospective nature of the data, which did not allow for a detailed evaluation of the timing and treatment of these conditions. Future studies with longitudinal follow-ups and more comprehensive clinical histories and treatment data will be essential to clarify the potential impacts of these comorbidities on cognitive decline.

Depression emerged as a significant predictor of cognitive decline, affecting both the disease onset and progression. Over 70% of the patients in our study had some form of depression, with moderate and severe depression showing strong associations with lower MMSE scores and accelerated cognitive deterioration. The severity of depression, as assessed using the MADRS and Hamilton scales, revealed a dose–response relationship, where greater depressive symptoms correlated with more pronounced cognitive deficits. This aligns with previous findings that major depression plays a crucial role in AD progression, with prevalence rates ranging from 5% to as high as 85% in AD patients, depending on diagnostic criteria [47,48].

Several studies have highlighted that patients with AD and concurrent depression experience faster declines in memory, executive function, and daily living skills compared to those without depression. This is supported by findings indicating that depression in AD is associated with increased neuroinflammation, dysregulation of neurotransmitters, and greater amyloid-β accumulation [49,50]. The bidirectional relationship between depression and AD risk further reinforces the need for integrated psychiatric and neurological care. Patients with recent depressive episodes were found to have nearly twice the risk of developing AD compared to those without a history of depression, suggesting that mood disorders may be both a symptom and a contributing factor in neurodegeneration.

Given the strong association between depression and cognitive impairment, early screening and treatment for depression should be a key part of AD management. Targeted interventions, including cognitive–behavioral therapy and pharmacological treatments, may help to mitigate cognitive decline, improve quality of life, and slow disease progression in AD patients with comorbid depression [51].

Although several predictors, like age, depression severity, and gender, showed statistically significant associations with MMSE scores, the magnitude of their effects was generally modest from a clinical perspective. For example, reductions of 1 to 2.7 points in MMSE scores across depression stages, or a 2.6-point difference between the sexes, are unlikely to reflect substantial impairment in daily functioning on their own. These values should be interpreted cautiously and within the broader context of natural cognitive variability in AD. However, we also emphasize that clinical significance cannot always be inferred from average group effects. The substantial random effects observed in the mixed models point to meaningful within-group heterogeneity, implying that for some patients, the impacts of factors such as depression or age may be considerably more pronounced. This observation supports the growing emphasis on personalized medicine and justifies the choice of multilevel modeling in our analysis, which accounts for inter-individual variability in disease progression.

Anti-AD therapies were analyzed to assess their impacts on MMSE scores. The findings indicate that MMSE ratings declined over time in all the groups, independent of the therapeutic approach. Patients who did not receive any therapy began with higher scores and saw modest drops, whereas those who received treatments had lower ratings from the beginning. This shows that therapy began later in the disease’s progression. The donepezil and memantine + rivastigmine groups had the greatest declines, but the confidence intervals overlapped, indicating that the differences between the treatments were not statistically significant. When interpreting the evolution of MMSE scores across treatment groups, it is important to consider that patients receiving pharmacological therapies, especially combination regimens, started from lower cognitive baselines. This suggests that treatments were more frequently prescribed in more advanced stages of the disease. Consequently, the differences in cognitive decline between the therapy groups may partly reflect baseline disparities rather than pure treatment effects. In other words, it is difficult to tell for sure which therapy was more beneficial than another. These findings support the idea that AD is progressive and that existing therapies have limited impacts on the rate of the cognitive deterioration. Interventions must be initiated earlier and more specifically for each patient.

A 2023 review analyzing forty-three AD clinical trials from 2015 to 2022 found that most treatments were ineffective, with only seven studies reporting both safety and therapeutic benefits. Three trials showed toxicity despite therapeutic effects [52]. Another review of 149 studies concluded that *Ginkgo biloba*, Cerebrolysin, and AChE inhibitors (donepezil, galantamine, rivastigmine, and huperzine A) may improve cognitive function and daily activities, but anti-Aβ drugs showed limited efficacy in slowing cognitive decline [53]. Corroborating all these, it is important to emphasize the critical role of therapy in managing AD, underscoring the need for early and tailored interventions to optimize patient outcomes.

The MADRS scale indicates a strong correlation between depression severity and cognitive function (*p* < 0.001); as the severity of AD increases (from mild to severe), the reduction in depressive symptoms becomes more substantial and statistically significant. Both moderate and severe stages of AD show highly significant negative associations with MADRS scores. For the Hamilton scale, the relationship between disease severity and depressive symptoms is less clear. Only the severe stage (SIV) showed a statistically significant reduction in Hamilton scores, while the mild and moderate stages brought no significant changes in depressive symptoms, as measured based on the Hamilton scale. These results underscore the detrimental impact of untreated or poorly managed depression on AD progression and emphasize the importance of addressing mental health in this population [54]. In this study, both MADRS and Hamilton scales were used to assess depression severity in patients with AD. Although both are validated instruments in dementia populations, they differ conceptually in the symptom dimensions they emphasize. The MADRS scale focuses primarily on cognitive–affective symptoms, such as sadness, con-centration difficulties, and emotional reactivity, whereas the Hamilton depression rating scale (HDRS) incorporates more somatic- and anxiety-related items, such as insomnia and gastrointestinal symptoms.

This distinction may partly explain the divergent findings observed in our analysis, where MADRS demonstrated a stronger and more consistent association with cognitive performance (MMSE, Reisberg, and clock scores), especially at moderate and severe levels of depression. In contrast, only the most severe stage of depression measured based on the Hamilton scale was significantly associated with cognitive scores. A Spearman correlation analysis between the two scales at the baseline further supported this divergence, revealing a weak and statistically non-significant correlation (ρ = 0.106, *p* = 0.289), suggesting that the scales may not be interchangeable in the context of cognitive decline.

These findings are in line with previous research, indicating that MADRS may be more sensitive in detecting depression in dementia, particularly because of its focus on affective–cognitive symptoms rather than somatic complaints, which can overlap with symptoms of AD itself (e.g., fatigue and sleep disturbance). For instance, a study on 89 patients with early-onset dementia (EOD) found that MADRS was effective in identifying depressed from non-depressed EOD individuals and showed strong congruence validity in evaluating depression symptoms. The conclusion was that MADRS intensity grades can be used to construct or modify depression measures in (early-onset) dementia [55].

Quilty et al. found that MADRS was responsive in detecting clinically meaningful changes in mood in AD patients, like sadness, negative thoughts, detachment, and neurovegetative symptoms. This four-factor structure remained consistent across time and gender, evidence supporting the use of the MADRS total score and subscales focused on affective, cognitive, social, and physical components of depression in outpatients [23].

It is also important to note that although this was a single-center study, assessments were performed by multiple clinicians, and potential inter-rater variability may have contributed to the observed inconsistencies. This reinforces the need for standardized training and possibly the prioritization of instruments less sensitive to inter-rater variability.

This study also underscores the heterogeneous nature of AD progression and suggests the need for personalized approaches to care through the results of the random-effect model, which showed significant differences between patients when considering the MMSE score. These results validate the analytical approach and provide a reliable basis for interpreting the predictors’ impact.

The MADRS and HAM-D scales remain valuable instruments for assessing depression in AD patients. Recent studies have suggested that although these scales are effective, their performance can be influenced by disease-specific factors, and their sensitivity and specificity may vary. Therefore, clinicians should consider these factors when selecting and interpreting depression assessment tools in the context of AD [56].

The Bayesian models used in this research have shown significant utility in complex longitudinal studies, and the application of larger and better-calibrated datasets could help to improve the precision of estimates in the future. The results of this study indicate progressive cognitive deterioration, according to the Reisberg scale, in patients over time. The intercepts showed increasingly positive scores as the disease advances, which correspond to worsening dementia. Similarly, the time-effect comparison (at Moments II and III) demonstrated that cognitive decline accelerates over time. The pattern of progressive cognitive deterioration and the accelerating decline in later stages match with those in recent research using similar methods, like Bayesian modeling. These studies reinforce the idea that AD follows a predictable pattern of worsening cognitive function, especially in later stages, because of the cumulative effects of neurodegeneration and that time-dependent accelerations in cognitive decline are characteristic of the disease’s trajectory [57,58].

In analyzing the predictors affecting the Reisberg score’s progression (age, sex, and various rating scales, such as the MADRS and Hamilton depression scale), the intercepts at various stages indicate a steady increase in cognitive decline as the disease progresses. The stage estimates (especially at stages 4–5, 5–6, and 6–7) indicated more substantial cognitive impairment, which aligns with the expected pattern in AD. Neither age nor sex had any significant effect on cognitive decline progression in this model, which might suggest that other factors (e.g., depression and comorbid conditions) may play larger roles in cognitive decline progression and that both sexes, in this sample, may experience similar rates of cognitive decline. The MADRS scores, particularly at moderate and severe stages, significantly predict cognitive decline, supporting, once more, the idea that depression (as measured based on MADRS) is a relevant factor influencing cognitive deterioration. Conversely, Hamilton scales show mixed results, with some stages showing potential effects but not as consistently as MADRS scores. Studies before have reinforced the importance of utilizing depression-rating scales not only for assessing mood disorders but also for monitoring cognitive health, particularly in populations at risk for depression-related cognitive decline [59].

In estimating the coefficients and credibility intervals for predictors of the Reisberg score and the effects of therapies, the results of this study showed that as AD progresses, cognitive decline becomes more pronounced, particularly in stages 1–2, 2–3, and 6–7, where the cognitive scores become increasingly negative. However, at stages like 3–4 and 4–5, there is uncertainty or potential for a plateau in the decline. The effects of therapies across different stages (from CI to CV) are generally positive but with no statistically significant *p*-value. The wide credibility intervals for each therapy stage suggest uncertainty about the effectiveness of the interventions in altering the Reisberg score. The therapies used in this study did not show any statistically significant improvements in slowing cognitive decline, suggesting that further research and larger samples may be needed to confidently establish the efficacy of these treatments. Clinicians should be cautious when interpreting the effects of different therapies, as the uncertainty in the results may imply that more robust or targeted interventions are necessary to effectively influence cognitive outcomes. These results are in line with those in other research, which underlines the need for further validation of the long-term efficacy of the anti-AD drugs [60]. Not even the anti-amyloid therapies showed any important evidence in slowing the progressions of the disease and the cognitive decline [61]. Thus, further research using larger cohorts and more precise longitudinal designs is needed to validate therapeutic impacts on functional outcomes.

The Bayesian model was also applied for additional analysis of the evolution of the clock test scores. The results indicate that cognitive function tends to worsen progressively and that interventions or additional factors might need to be addressed during the early stages to potentially slow the decline. The relatively clear and significant findings at Moment II and Moment III suggest that interventions may be necessary in these periods to counteract further cognitive deterioration. In investigating the predictors of the clock test, the outcomes of this study showed that age did not appear to have a strong effect on cognitive performance and that the male gender was associated with better cognitive performance. The same results were found in larger cohort studies [62].

As for the depression severity, as indicated by MADRS and Hamilton stages, it had varying effects on the clock score. In general, Stage I of both scales seems to correlate with mild improvements or neutral effects, while higher stages (II and III) tend to be associated with cognitive decline, though with substantial uncertainty. These results are consistent with broader research trends published in the last few years [63]. However, the observed associations with affective symptoms showed wide credibility intervals, suggesting high variability and a need for cautious interpretation. Although the clock-drawing test remains a widely used screening tool for cognitive decline, its sensitivity to subtle longitudinal changes may be limited, particularly in early disease stages. This could partially explain the weaker predictive performance of this model compared to those based on MMSE and Reisberg scores. Future studies may consider combining clock test results with domain-specific cognitive tasks to better capture visuospatial deterioration and its interplay with psychiatric symptoms.

This study has several limitations that should be considered when interpreting the findings. First, the sample was relatively homogeneous, consisting mostly of older women from rural areas. Although this reflects the clinical population treated at our center, it may limit the generalizability of the results to other demographic groups, such as urban populations or younger patients. Second, the follow-up period of 12 months provides only a short-to-medium-term view of cognitive trajectories, without capturing the longer-term effects of disease progression or treatment. Third, the absence of biomarker data—such as neuroimaging or cerebrospinal fluid analysis—limits the ability to link observed cognitive changes to underlying biological mechanisms. Additionally, the retrospective nature of the dataset restricted our capacity to accurately assess the timing and evolution of comorbidities or treatments. Although our statistical models accounted for the independent effects of these factors, we did not investigate potential interactions between them, for instance, whether specific comorbidities might alter the effects of certain pharmacological therapies. This limitation was largely because of small subgroup sizes and the limited clinical granularity of the available data.

Despite these limitations, this study offers a comprehensive analysis of AD progression, integrating demographic, clinical, and psychological factors. The use of advanced statistical modeling, including random-effect and Bayesian analyses, enhances the robustness of the findings by accounting for individual variability in cognitive decline. Furthermore, the study population, composed of elderly women from rural areas, reflects real-world relevance, making the results highly applicable to similar patient groups. These findings highlight the multifaceted nature of AD progression, reinforcing the need for a personalized, multidisciplinary approach that incorporates neurological, psychiatric, and cardiovascular care to improve patient outcomes.

## 5. Conclusions

The findings of this study indicate that different factors influence the progression of AD, with age and depression playing important roles in cognitive decline. Most patients received memantine-based therapy, particularly in the intermediate and severe stages; however, AD follows a predictable decline in cognition, especially in later stages, so medication response should be interpreted cautiously. Depression was frequently encountered and was clearly correlated with faster deteriorations in memory and daily functioning, which emphasizes the importance of assessing and adequately treating affective symptoms. Although comorbidities, such as hypertension and diabetes, were prevalent, they had no significant impacts on cognitive performance in the studied population. The integrated evaluation of cognitive and emotional statuses, along with the use of modern statistical models, supports the idea that AD patients require a tailored strategy that considers not only the stage of the illness but also the unique characteristics of each patient.

## Figures and Tables

**Table 1:** Standardized tests designed for monitoring cognitive performance and depression symptoms.

Type of Test/Scale	Description	Evaluation/Scores	Ref.
MMSE (Mini-mental state examination)	It uses exercises and questions to evaluate cognitive abilities; scores reveal the stage of cognitive decline. It involves evaluating language, memory, attention, and spatial and temporal orientations. The interpretation of the total score, which varies from 0 to 30 points, aids in identifying the stage of cognitive decline.	A score of less than 24 may indicate cognitive impairment.	[20]
Reisberg scale	It determines the severity of AD according to progressive cognitive decline in seven stages (from no decline to extremely severe decline). This scale is useful for determining the need for assistance based on the patient’s degree of cognitive impairment.	These stages range from no cognitive decline (Stage I) to extremely severe cognitive decline (Stage VII).	[21]
Clock test	It evaluates spatial orientation ability, being sensitive to the detection of cognitive decline. Patients draw a clock indicating a certain time. The patient is given a sheet of paper and has to draw a circle, fix the 12 o’clock position correctly and position the numbers on the circle. The patient must also trace the hands of the clock into the correct positions.	Each correct step is worth one point, and lower scores indicate cognitive difficulties.	[22]
MADRS (Montgomery–Åsberg depression rating scale)	It evaluates depression symptoms for ten items that measure many elements of depression, including sorrow, sleep, and concentration.	Scores range from normal (0) to VI, from mild to the most severe stages.	[23]
HAM-D (Hamilton rating depression scale)	It evaluates depression symptoms in detail. Twenty-one items that measure mood, anxiety, sleeplessness, and other depressive symptoms are included. Clinicians can use the results to customize treatment interventions and gain a detailed picture of the patient’s emotional state.	Although items 18 to 21 offer additional information not covered by the scale (such as paranoia and diurnal variation), the first 17 of the 21 items are used to determine the final score (the total score ranges from 0 to 52).	[24]

**Table 2:** Distribution of demographic and clinical characteristics of the participants.

Variable	*n*	Mean ± SD/ Percentage (%)
Age (years)	75	±9
Sex		
Female	78	77.23
Male	23	22.77
Environment		
Rural	57	56.44
Urban	44	43.56
Educational Level		
None	19	18.81
Primary	18	17.82
Secondary	64	63.37
Treatment		
Memantine	32	31.68
Donepezil, 10 mg	1	0.99
Donepezil, 20 mg	6	5.94
Rivastigmine, 4.6 mg	2	1.98
Memantine + Rivastigmine, 4.6 mg	11	10.89
Memantine + Rivastigmine, 9.5 mg	12	11.88
Memantine + Donepezil, 5 mg	9	8.91
Memantine + Donepezil, 10 mg	24	23.76
No Treatment	4	3.96

SD, standard deviation; *n*, number of individuals.

**Table 3:** Analysis of cognitive and psychological characteristics of patients with Alzheimer’s dementia.

Variable	Number of Subjects	Mean ± SD/ Percentage (%)
MMSE Score (Mean ± SD)	18.6	±5.6
Cognitive Impairment Severity (MMSE)		
Mild (MMSE > 20)	38	37.62
Moderate (MMSE 10–20)	44	43.56
Severe (MMSE < 10)	18	17.82
Reisberg Global Deterioration Scale (GDS)		
Stage 3—Mild Cognitive Impairment	10	9.90
Stage 4—Moderate Cognitive Decline	32	31.68
Stage 5—Moderate–Severe Decline	41	40.59
Stage 6–7—Severe Cognitive Decline	17	16.83
Clock		
Stage I—Normal Performance	17	16.83
Stage II—Mild Vasoconstrictive Impairment	42	41.58
Stage III—Moderate Vasoconstrictive Impairment	24	23.76
Stage IV—Severe Vasoconstrictive Impairment	18	17.82
MADRS		
Normal—Absence of Depression	29	28.71
Stage I—Mild Depression	38	37.62
Stage II—Moderate Depression	16	15.84
Stage III—Severe Depression	18	17.82
Hamilton		
Stage I—Mild Depression	2	27.72
Stage II—Moderate Depression	31	30.69
Stage III—Severe Depression	34	23.76
Stage IV—Very Severe Depression	8	7.92

SD, standard deviation; *n*, number of individuals, MMSE, mini-mental state examination; MADRS, Montgomery–Åsberg depression rating scale.

**Table 4:** MMSE scores’ marginal means throughout the three measurement times.

Marginal Mean				
			95% CI	
Moment	Estimate	ES	Lower Limit	Upper Limit
TI	18.564	0.559	17.469	19.659
TII	17.673	0.559	16.578	18.768
TIII	16.604	0.559	15.509	17.699

CI, confidence interval; ES, standard error.

**Table 5:** Mixed-effect model’s results for MMSE scores.

Predictor	Observations, *n*	Beta (95% CI)	*p*-Value
Moment			
T0	101	—	
T1	101	−0.89 (−1.3 to −0.45)	<0.001
T2	101	−2.0 (−2.4 to −1.5)	<0.001
Age	303	−0.13 (−0.25 to −0.02)	0.026
Sex			
F	234	—	
M	69	2.6 (0.03 to 5.1)	0.047
Environment			
Rural	171	—	
Urban	132	−0.13 (−2.3 to 2.1)	0.903
Educational Level			
None	57	—	
Primary School	54	1.1 (−2.5 to 4.6)	0.545
High School	192	−1.3 (−4.1 to 1.5)	0.372
Type of Diagnosis			
Principal	228	—	
Secondary	75	1.5 (−0.97 to 4.0)	0.228

CI, confidence interval; F, female; M, male.

**Table 6:** The impacts of medical conditions on Alzheimer’s disease patients’ MMSE scores.

Predictor	Observations, *n* (Yes/No)	Beta (95% CI)	*p*-Value
Brain Atrophy	57/246	−0.71 (−3.5 to 2.1)	0.611
Anemia	27/276	3.4 (−0.35 to 7.1)	0.075
Diabetes Mellitus	63/240	−1.3 (−4.0 to 1.3)	0.331
Polyneuropathy	6/297	−5.8 (−14 to 1.9)	0.136
Thrombocytopenia	12/291	3.9 (−1.6 to 9.4)	0.159
Atrial Fibrillation	21/282	−0.62 (−4.9 to 3.6)	0.774
Chronic Kidney Disease	18/285	−2.2 (−6.7 to 2.4)	0.345
Hypertension Stage I	72	1.6 (−1.3 to 4.5)	0.275
Hypertension Stage II	117	1.2 (−1.3 to 3.7)	0.354
Hypertension Stage III	9	−3.0 (−9.5 to 3.5)	0.367
Dyslipidemia	36/267	−2.8 (−6.1 to 0.53)	0.099
Epilepsy	9/294	−1.8 (−8.1 to 4.6)	0.583
Obesity Stage I	9	3.8 (−2.6 to 10)	0.241
Obesity Stage II	18	1.1 (−3.5 to 5.7)	0.628
Heart Failure Stage II	21	1.1 (−3.2 to 5.4)	0.600
Heart Failure Stage III	15	−0.84 (−5.9 to 4.2)	0.740

CI, confidence interval; *p*, statistical significance; LVH, left ventricular failure; CHF, congestive heart failure; DM, diabetes mellitus; HTA, hypertension; AF, atrial fibrillation.

**Table 7:** Predictors of MADRS and Hamilton scores in Alzheimer’s dementia: estimates and statistical values.

Predictor	Observations, *n*	Beta (95% CI)	*p*-Value
MADRS			
Normal	63	—	
SI—Mild	110	−1.1 (−2.1 to −0.13)	0.027
SII—Moderate	71	−2.0 (−3.2 to −0.87)	<0.001
SIII—Severe	59	−2.7 (−4.1 to −1.4)	<0.001
Hamilton			
SI	64	—	
SII	93	−0.05 (−1.0 to 0.92)	0.922
SIII	100	−0.74 (−1.9 to 0.47)	0.231
SIV	46	−2.2 (−3.5 to −0.77)	0.002

CI, confidence interval; SI, stage I; SII, stage II; SIII, stage III; SIV, stage IV; *p*, statistical significance; MADRS, Montgomery–Åsberg depression rating scale.

**Table 8:** Marginal means of MADRS scores according to depression severity.

Marginal Mean				
Variable	Estimate	ES	95% CI	
			Lower Limit	Upper Limit
MADRS				
Normal	19.026	0.666	17.720	20.332
SI	17.910	0.593	16.749	19.072
SII	17.012	0.629	15.778	18.245
SIII	16.278	0.683	14.940	17.616
Hamilton				
SI	18.199	0.674	16.877	19.521
SII	18.151	0.619	16.937	19.365
SIII	17.464	0.618	16.252	18.675
SIV	16.040	0.699	14.670	17.410

SE, standard error; CI, confidence interval; SI, stage I; SII, stage II; SIII, stage III.

**Table 9:** Analysis of the effectiveness of drugs in the treatment of Alzheimer’s disease.

Predictor	*n* (Yes/No)	Beta (95% CI)	*p*-Value
Memantine	264/39	0.38 (−2.9 to 3.6)	0.814
Donepezil (Dose I)	30/183	−2.4 (−6.1 to 1.3)	0.194
Donepezil (Dose II)	90/183	−0.51 (−2.9 to 1.9)	0.680
Rivastigmine (Dose I)	39/228	−1.7 (−5.0 to 1.6)	0.305
Rivastigmine (Dose II)	36/228	−1.2 (−4.6 to 2.1)	0.472
Antidepressants	183/120	−0.84 (−3.0 to 1.4)	0.454
Antiepileptics	54/249	−1.4 (−4.2 to 1.4)	0.329
Antiparkinsonian Drugs	12/291	−3.4 (−9.0 to 2.1)	0.219
Antipsychotics	129/171	−0.62 (−2.8 to 1.6)	0.579
Anxiolytics	177/126	−0.72 (−2.9 to 1.5)	0.514
Sedatives/Hypnotics	84/219	0.01 (−2.4 to 2.4)	0.994
Psychostimulants	15/288	3.2 (−1.7 to 8.2)	0.197
Vasoprotectors	9/294	−2.0 (−8.4 to 4.4)	0.535

CI, confidence interval; *p*, statistical significance.

**Table 10:** Evolution of MMSE scores at the three evaluation moments, depending on the therapy.

Therapy	Moment	MMSE	95% CI		*p*-Value
			Lower Limit	Upper Limit	
No Therapy	T0	23.750	18.220	29.280	0.085
	T1	23.083	17.790	28.376	
	T2	21.750	16.223	27.277	
Memantine (CI)	T0	19.406	17.451	21.362	0.116
	T1	18.542	16.67	20.413	
	T2	17.687	15.733	19.642	
Donepezil (CII)	T0	17.286	13.105	21.466	0.026
	T1	15.429	11.427	19.430	
	T2	13.714	9.536	17.892	
Rivastigmine (CIII)	T0	16.500	8.679	24.321	0.163
	T1	16.500	9.015	23.985	
	T2	15.500	7.684	23.316	
Memantine + donepezil (CIV)	T0	18.303	16.378	20.228	0.048
	T1	17.354	15.511	19.196	
	T2	16.394	14.47	18.318	
Memantine + rivastigmine (CV)	T0	17.435	15.128	19.741	0.027
	T1	16.507	14.3	18.715	
	T2	15.478	13.173	17.783	

CI, confidence interval; *p*, statistical significance.

**Table 11:** Demographic, clinical, and therapeutic predictors associated with MMSE scores (mixed model).

Predictor	Beta	95% CI	*p*-Value
Age	−0.09	−0.22 to 0.04	0.170
Sex			
Female	Reference	-	-
Male	1.10	−1.80 to 4.00	0.440
MADRS (Depression Severity)			
Normal	Reference	-	-
Mild (SI)	−1.10	−2.00 to −0.07	0.035
Moderate (SII)	−1.70	−2.90 to −0.58	0.003
Severe (SIII)	−2.50	−3.80 to −1.10	<0.001
Hamilton (Anxiety Severity)			
Stage I (SI)	Reference	-	-
Stage II (SII)	0.25	−0.71 to 1.20	0.610
Stage III (SIII)	−0.51	−1.70 to 0.68	0.400
Stage IV (SIV)	−1.70	−3.10 to −0.35	0.014
Therapy			
No Therapy	Reference	-	-
Memantine (CI)	−3.60	−9.40 to 2.20	0.220
Donepezil (CII)	−6.50	−13.00 to 0.33	0.062
Rivastigmine (CIII)	−7.30	−16.00 to 1.90	0.120
Memantine + donepezil (CIV)	−5.10	−11.00 to 0.70	0.084
Memantine + rivastigmine (CV)	−6.20	−12.00 to −0.38	0.037

CI, confidence interval; MADRS, Montgomery–Åsberg depression rating scale; SI, SII, SIII, and SIV, stages of depression or anxiety severity; MMSE, mini-mental state examination.

**Table 12:** Impact of both age and depression severity on MMSE scores.

Predictor	Observations (*n*)	Beta (95% CI)	*p*-Value
Age	303	−0.12 (−0.24 to 0.00)	0.041
MADRS			
Normal	63	—	—
Mild	110	−1.1 (−2.1 to −0.11)	0.030
Moderate	71	−2.0 (−3.1 to −0.83)	<0.001
Severe	59	−2.7 (−4.0 to −1.4)	<0.001

MADRS, Montgomery–Åsberg depression rating scale; CI, confidence interval; *n*, number of observations; *p*, statistical significance.

**Table 13:** Bayesian model results: estimates of intercepts and time effects for the Reisberg score progression.

Predictor	Mean	95% CI	
		Lower Limit	Upper Limit
Intercept (1–2)	−11.44	−13.92	−9.21
Intercept (2–3)	−5.52	−6.98	−4.16
Intercept (3–4)	−1.34	−2.59	−0.12
Intercept (4–5)	2.99	1.71	4.34
Intercept (5–6)	5.74	4.37	7.28
Intercept (6–7)	9.97	8.13	11.97
Moment II	0.65	0.07	1.25
Moment III	2.14	1.49	2.83

Thresholds (Intercepts) indicate transition points between successive levels of Alzheimer’s severity based on the Reisberg scale. Estimates reflect the posterior means and 95% credible intervals from the Bayesian ordinal regression model. CI = Confidence Interval.

**Table 14:** Estimation of coefficients and credibility intervals for predictors of the Reisberg score according to age, sex, and various rating scales.

Predictor	Mean Estimate	95% CI	
		Lower Limit	Upper Limit
Intercept (1–2)	−3.11	−12.79	6.72
Intercept (2–3)	2.45	−7.12	12.22
Intercept (3–4)	6.26	−3.2	16.09
Intercept (4–5)	10.32	0.82	20.36
Intercept (5–6)	12.79	3.19	22.93
Intercept (6–7)	16.67	7.02	26.96
Age	0.1	−0.02	0.23
Sex (Male)	−1.16	−3.47	1.05
MADRS Stage I	0.5	−0.59	1.58
MADRS Stage II	1.45	0.2	2.73
MADRS Stage III	1.65	0.23	3.12
Hamilton Stage II	−0.37	−1.46	0.73
Hamilton Stage III	0.66	−0.59	1.93
Hamilton Stage IV	1.19	−0.23	2.65

CI, confidence interval.

**Table 15:** Estimation of coefficients and credibility intervals for predictors of the Reisberg score and the effects of therapies.

Predictor	Mean	95% CI	
		Lower Limit	Upper Limit
Intercept (1–2)	−11.03	−14.05	−8.21
Intercept (2–3)	−5.58	−7.78	−3.35
Intercept (3–4)	−1.9	−4.05	0.2
Intercept (4–5)	1.9	−0.22	4.07
Intercept (5–6)	4.29	2.08	6.51
Intercept (6–7)	8	5.63	10.5
Therapy CI	−0.78	−3.24	1.62
Therapy CII	0.92	−2.13	4.12
Therapy CIII	0.62	−3.15	4.67
Therapy CIV	0.33	−2.02	2.76
Therapy CV	0.82	−1.63	3.32

**Table 16:** Model performance indicators and predictor contribution (Bayesian LOOCV).

Model	elpd_loo	SE elpd	p_loo	LOOIC	Δ ELPD vs. Full	SE Δ ELPD
Full model (all the predictors)	−287.5	13.6	84.5	575.0	0.0	0.0
Only MADRS	−290.5	—	—	—	−3.0	2.0
3 predictors	−290.8	—	—	—	−3.3	1.9
2 predictors	−291.1	—	—	—	−3.6	1.9

LOOIC, leave-one-out information criterion; elpd_loo, expected log pointwise predictive density; p_loo, effective number of parameters; SE, standard error.

**Table 17:** Estimates of coefficients and credibility intervals for the model predictors.

Predictor	Mean	95% CI	
		Lower Limit	Upper Limit
Intercept (1)	−4.46	−5.93	−3.18
Intercept (2)	0.25	−0.95	1.43
Intercept (3)	5.2	3.77	6.81
Moment II	−1.15	−1.86	−0.45
Moment III	−1.38	−2.13	−0.67

**Table 18:** Estimates of coefficients and credibility intervals for predictors of the clock score.

Predictor	Mean	95% CI	
		Lower Limit	Upper Limit
Intercept (1–2)	−2.63	−12.3	6.54
Intercept (2–3)	1.88	−7.68	11.07
Intercept (3–4)	6.62	−2.97	15.97
Age	0	−0.13	0.12
Sex (M)	3.32	0.93	5.88
MADRS SI	0.61	−0.55	1.8
MADRS SII	−0.13	−1.44	1.17
MADRS SIII	−0.54	−2.11	0.99
Hamilton SII	0.63	−0.56	1.85
Hamilton SIII	−0.08	−1.45	1.31
Hamilton SIV	−0.78	−2.4	0.84

Intercepts represent estimated thresholds between adjacent levels of the ordinal clock score. For example, Intercept (1–2) reflects the boundary between scores 1 and 2.

**Table 19:** Model performance indicators and predictor contributions based on LOOCV.

Model	elpd_loo	SE elpd	p_loo	LOOIC	Δ ELPD vs. Only Sex	SE Δ ELPD
Full model (all the predictors)	−228.4	13.6	86.9	456.7	−4.4	2.6
Only sex	−224.0	12.8	78.1	448.0	0.0	0.0

ELPD, expected log pointwise predictive density; LOOIC, leave-one-out information criterion; p_loo, effective number of parameters; SE, standard error.

## Data Availability

The data supporting the reported results can be found in the archive of Bihor County Emergency Clinical Hospital in Oradea, Romania, where the data of the patients are registered.

## Abbreviations

The following abbreviations are used in this manuscript:

Abbreviation	Full Term
AD	Alzheimer’s disease
MMSE	mini-mental state examination
GDS	global deterioration scale (Reisberg scale)
MADRS	Montgomery–Åsberg depression rating scale
HAM-D/HDS	Hamilton depression scale
CI	confidence interval
SD	standard deviation
ICC	intraclass correlation coefficient
R^2^	coefficient of determination
TI, TII, TIII	evaluation time points
SI, SII, SIII, SIV	depression or anxiety severity stages
CI, CII, etc.	Treatment groups (e.g., CI = memantine and CII = donepezil)
R	R programming environment
AChE	acetylcholinesterase
NMDA	N-methyl-D-aspartate
